# Lagrange Interpolation Learning Particle Swarm Optimization

**DOI:** 10.1371/journal.pone.0154191

**Published:** 2016-04-28

**Authors:** Zhang Kai, Song Jinchun, Ni Ke, Li Song

**Affiliations:** Mechanical Engineering and Automation, Northeast University, Shenyang, China; Beihang University, CHINA

## Abstract

In recent years, comprehensive learning particle swarm optimization (CLPSO) has attracted the attention of many scholars for using in solving multimodal problems, as it is excellent in preserving the particles’ diversity and thus preventing premature convergence. However, CLPSO exhibits low solution accuracy. Aiming to address this issue, we proposed a novel algorithm called LILPSO. First, this algorithm introduced a Lagrange interpolation method to perform a local search for the global best point (gbest). Second, to gain a better exemplar, one gbest, another two particle’s historical best points (pbest) are chosen to perform Lagrange interpolation, then to gain a new exemplar, which replaces the CLPSO’s comparison method. The numerical experiments conducted on various functions demonstrate the superiority of this algorithm, and the two methods are proven to be efficient for accelerating the convergence without leading the particle to premature convergence.

## 1. Introduction

In 1995, Kennedy and Elberhart, inspired by the foraging behaviour of birds, proposed the particle swarm optimization (PSO) algorithm [1], which has attracted attention in the academic circles and has demonstrated its superiority in solving practical problems. PSO is a type of evolutionary algorithm, which is similar to the simulated annealing (SA) [2] algorithm. PSO starts from a random solution, searches for the optimal solution in iterative manner, and then evaluates the quality of the solution based on the fitness. PSO follows the current optimal value to find the global optimum, so it is simpler than the genetic algorithm (GA) [3], without the need for the “cross” and “mutation” operations. However, PSO suffers two problems: premature and slow convergence at the late stage. In the past two decades, many researchers have focused on addressing these problems by introducing some methods and concepts. A concept of inertia weight was introduced and applied in the formula by Y.Shi and Eberhart, they set the weight from 0.9 to 0.4 to provide a balance between exploitation and exploration [4][5]; On the basis of that, later researchers developed adaptive inertia weight and coefficients [6][7]. To avoid the premature convergence of the particle swarm, Riget J and Vesterstrim Js proposed a concept of diversity: they set a lower bound of diversity to ensure the swarm has a good search ability [8]. A simulated annealing (SAPSO) idea was introduced to help a particle jump out of the local [9]. A grey relational analysis was introduced for changing the parameters of PSO to help improve the algorithm performance in [10]. A chaotic search idea to global search was proposed in [11], which was improved by introducing the sequence quadratic program (SQP) algorithm to accelerate convergence in [12]. The gradient search for accurate computation of the global minimum was proposed in [13]. Meanwhile, Some researchers focus on structural and heterogeneous factor, such as SIPSO [14], SFPSO [15] and LIPSO [16].

To solve the multimodal problems, J.J. Liang proposed the comprehensive learning particle swarm optimizer (CLPSO) [17]. Later, Liang and Suganthan [18] proposed an adaptive CLPSO with historical learning, for which the particles’ learning probabilities are adjusted adaptively. On the basis of that finding, researchers realised that CLPSO’s search method was quite efficient for finding the global optimum. However, CLPSO suffers a slow resolution. Aiming to this point, some improved CLPSO was proposed. An orthogonal experimental design was introduced in CLPSO to determine the best combination of learning from a particle’s personal best position or its neighbourhood’s historical best position [19]. Zheng et al. [20] proposed ACLPSO which adaptively sets the factors of the algorithm, i.e. the inertia weight and acceleration coefficient. Nasir [21] proposed a DNLPSO which used a learning strategy, whereby all other particles’ historical best information was used to update a particle’s velocity, as in CLPSO. However, in contrast to CLPSO, the exemplar particle was selected from a neighbourhood. The neighbourhoods were made dynamically in nature, i.e, they are reformed after certain intervals. Xiang Yu [22] introduced a kind of perturbation in to the iterative forms of the CLPSO. It determined the particles’ learning probabilities between it’s historical best values and the dimensional bounds. Because there is a bound for the perturbation, it will speed up the convergence. On the base of CLPSO, some multi-objective optimization problems can be solved by using Pareto dominance concept [23][24][25].

In this research, we proposed a novel improved algorithm, called LILPSO, which was based on CLPSO by introducing the Lagrange interpolation method. There are two main differences between LILPSO and CLPSO. First, When this algorithm performs as CLPSO’s search method for some times, it will introduce one Lagrange interpolation computation for each dimension of the best point (gbest). This is a local search method, and it will help accelerating the convergence. Second, CLPSO selects the better one of the other two particles’ historical optimum (pbest) as the exemplar at the *d*^*th*^ dimension. Compared with CLPSO, LILPSO selects three points, which are the *i*^*th*^ particles’ historical optimum, another random particles’ historical optimum and the global optimum (*gbest*), to perform the Lagrange interpolation, and then to obtain a parabola, whose optimum is the exemplar we expected.

The remainder of this article is organized as follows: Section 2 provides related works regarding PSO and CLPSO, and discusses the Lagrange interpolation theory. In Section 3, the proposed LILPSO is discussed in sufficient detail. Section 4 provides the experimental results on different functions to prove the superiority of LILPSO. Section 5 presents the paper’s conclusions.

## 2. Related Works

### 2.1 PSO algorithm

Assuming the optimization problem is

$$\min f\left(x\right)=f\left(x_{1},x_{2}\cdots ,x_{n}\right)$$

$$s.t.\;\;\;\;x_{i}\in \left[L_{i},U_{i}\right]\;\;\;\;i=1,2,\cdots n$$

If the particle is denoted as *X*_*i*_ = (*x*_*i*1_, *x*_*i*2_, ⋯, *x*_*iD*_), Then the best position it experienced (the best fitness value) is *P*_*i*_ = (*p*_*i*1_, *p*_*i*2_, ⋯, *p*_*iD*_), also denoted by *p*_*best*_, The index of the best position experienced in particle group represented by symbol *g* is denoted by *Pg*, or *g*_*best*_. The speed of particle *i* is denoted by *V*_*i*_ = (*v*_*i*1_, *v*_*i*2_, ⋯, *v*_*iD*_), for each generation, its *d*^*th*^ dimension iteration functions are:

$$\begin{array}{c} v_{id}\left(t+1\right)=\omega v_{id}\left(t\right)+c_{1}r_{1}\left(p_{id}\left(t\right)-x_{id}\left(t\right)\right)+c_{2}r_{2}\left(p_{gd}\left(t\right)-x_{id}\left(t\right)\right) \end{array}$$ (1)

$$\begin{array}{c} x_{id}\left(t+1\right)=x_{id}\left(t\right)+v_{id}\left(t+1\right) \end{array}$$ (2)

Where, *c*_1_ and *c*_2_ are all positive constants, called learning factors; *r*_1_ and *r*_2_ are random numbers, which are from 0 to 1; *v*_*id*_ is the -dimension speed of each particle. The first part of the right of the equal sign in Eq 1 is caused by the particle previous velocity, is called “inertia” part. The second part is “cognition” part, which illustrates that the particle thinks itself, as well as influences the particle information itself of the next step. The third part is the “social” part, which illustrates the information shared and mutual cooperation, as well as the influences on the swarm information of the next step.

### 2.2 CLPSO algorithm

CLPSO iteration function is different from the standard PSO.

$$\begin{array}{c} v_{id}\left(t+1\right)=\omega v_{id}\left(t\right)+c_{1}r_{1}\left(p_{id}{\left(t\right)}^{\prime }-x_{id}\left(t\right)\right) \end{array}$$ (3)

$$\begin{array}{c} x_{id}\left(t+1\right)=x_{id}\left(t\right)+v_{id}\left(t+1\right) \end{array}$$ (4)

where, $\omega =\omega_{max}-\frac{t}{max\_gen}\left(\omega_{max}-\omega_{min}\right)$, *ω*_*max*_ = 0.9 and *ω*_*min*_ = 0.4. *p*_*id*_(*t*)′ is the exemplar of the *i*^*th*^ particle in the *d*^*th*^ dimension. If the *i*^*th*^ particle does not update its historical optimum (*pbest*) continuously and over the gap m (usually m = 7), then a random number from 0 to 1 will be generated; for each dimension, if this random number is less than *pc*(*i*)(Eq 5), then another two particles’ historical optimum values will be compared, with the better one chosen for the exemplar in the *d*^*th*^ dimension. If all the exemplars of a particle are its own *pbest*, then we will randomly choose one dimension to learn from another particle’s *pbest*’s corresponding dimension.

$$\begin{array}{c} pc\left(i\right)=0.05+0.45*\frac{\exp(10*(i-1)/(N-1))-1}{\exp(10)-1} \end{array}$$ (5)

### 2.3 Lagrange interpolation

The theory of Lagrange interpolation is to use a polynomial to represent the relationship between a number of things. For example, when observing a physical quantity, if we gain some different values at different places, then a polynomial can be simulated by Lagrange interpolation method. The aim of this method is mainly used for data fitting in engineering experiments. It is a kind of curve smoothly fitting method.

Generally, if the fitness *y*_0_, *y*_1_⋯*y*_*n*_ at n+1 points *x*_0_, *x*_1_⋯*x*_*n*_ of function *y* = *f*(*x*) are known, then a polynomial *y* = *P*_*n*_(*x*) is considered, who occupies the n+1 points and whose number is no less than n.

s.t.*P*_*n*_(*x*_*k*_) = *y*_*k*_, *k* = 0, 1, 2⋯*n*

If we want to estimate a point *ξ*, *ξ* ≠ *x*_*i*_, *i* = 0, 1, 2⋯*n*, then the fitness of *P*_*n*_(*ξ*) can be the approximate value of *f*(*ξ*) In this case, we obtain

$$\begin{array}{c} f\left(x\right)=\sum_{i=1}^{n}y_{i}P_{i}\left(x\right)+error,\;\;\left(i=1,2,\cdots n\right) \end{array}$$ (6)

Where,$error=\frac{f^{\left(n+1\right)}\left(\xi \right)}{(n+1)!}\omega_{n+1}\left(x\right)$, *ξ* is a parameter related with *x*, and *ω*_*n*+1_(*x*) = (*x* − *x*_0_)(*x* − *x*_1_)⋯(*x* − *x*_*n*_)

## 3. The proposed methods

### 3.1 Local search with Lagrange interpolation (LSLI)

The main idea of CLPSO’s search method is to make the particle experience all the local optima as much as possible. Hence, the Eq 3 of CLPSO cuts off the global optima part compared with the Eq 1 of PSO. Moreover, after learning from the other particle’s historical optima, CLPSO sets a gap value m to digest this information. It is no doubt that these procedure will slow the particle swarm convergence, but will be in favor of multimodal function solution.

To accelerate the convergence, we decide to add a kind of local search into CLPSO. By far, there are some kinds of efficient technique, i.e. sub-gradient [26][27][28], perturbation, mutation or chaotic search with neighborhood [29][30][31][32][33][34][35]. The technique of sub-gradient can find the convergence direction easily. However, for the discontinuous and non-differentiable problems, the direction obtained will mislead the convergence. In addition, the step size is hard to decide. The technique of perturbation with neighborhood is not influenced by the form of the function. However, this method has no convergence direction, and usually needs many additional function evaluations (FEs). Hence, we adapt the local search technique of Lagrange interpolation to weaken these problems.

For the *j*^*th*^ dimension of the *gbest*, we select three points to generate the information and perform Lagrange interpolation. One point is the *gbest* itself, another point and the last point are the perturbations nearby the *gbest*. The perturbation value is denoted by *delta*, shown in Eq 7.

$$\begin{array}{c} delta=rand*\eta *v(i,j) \end{array}$$ (7)

$$\begin{array}{c} \begin{array}{c} x_{0}\left(j\right)=gbest\left(j\right);\;\\ x_{1}\left(j\right)=gbest\left(j\right)+delta;\\ x_{2}\left(j\right)=gbest\left(j\right)-delta; \end{array} \end{array}$$ (8)

Where, *v*(*i*, *j*) is the particle’s speed who has the best fitness for each iteration; *η* is a very small coefficient. In this research, we set *η* = 0.5/*N*, *N* is the particle swarm size. In the *j*^*th*^ dimension space, the three points can generate a parabola by the Lagrange interpolation method, and the minimum point is desired. Eqs 9 and 10 is the Lagrange interpolation computation.

$$\begin{array}{c} f\left(x\right)=y_{0}\frac{\left(x-x_{1}\right)\left(x-x_{2}\right)}{\left(x_{0}-x_{1}\right)\left(x_{0}-x_{2}\right)}+y_{1}\frac{\left(x-x_{0}\right)\left(x-x_{2}\right)}{\left(x_{1}-x_{0}\right)\left(x_{1}-x_{2}\right)}+y_{2}\frac{\left(x-x_{0}\right)\left(x-x_{1}\right)}{\left(x_{2}-x_{0}\right)\left(x_{2}-x_{1}\right)} \end{array}$$ (9)

Where, *y*_0_ = *fitness*(*x*_0_), *y*_1_ = *fitness*(*x*_1_), *y*_2_ = *fitness*(*x*_2_). Let *I* = (*x*_0_ − *x*_1_)(*x*_1_ − *x*_2_)(*x*_2_ − *x*_0_), after calculating, we obtain a quadratic polynomial.

$$\begin{array}{c} \begin{array}{c} f\left(x\right)=ax^{2}+bx+c\\ a=\frac{1}{I}\left[\left(x_{2}-x_{1}\right)y_{0}+\left(x_{0}-x_{2}\right)y_{1}+\left(x_{1}-x_{0}\right)y_{2}\right]\\ b=-\frac{1}{I}\left[\left(x_{2}^{2}-x_{1}^{2}\right)y_{0}+\left(x_{0}^{2}-x_{2}^{2}\right)y_{1}+\left(x_{1}^{2}-x_{0}^{2}\right)y_{2}\right]\\ c=\frac{1}{I}\left[x_{1}x_{2}y_{0}\left(x_{2}-x_{1}\right)+x_{0}x_{2}y_{1}\left(x_{0}-x_{2}\right)+x_{0}x_{1}y_{2}\left(x_{1}-x_{0}\right)\right] \end{array} \end{array}$$ (10)

**Fig 1:**
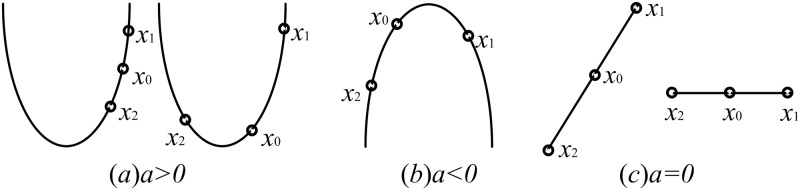
For*I* ≠ 0, the different cases of the solution.

Fig 1 shows the different cases of the desired solution. When *I* ≠ 0, which means the three points are different, if *a* > 0, then two cases will happen: one is that the *gbest* is between *x*1 and *x*2, and another is that the *gbest* is on one side of *x*1 and *x*2. At this time, we will choose the minimum point -b/2a as the solution, then compare it with the *gbest*. If *a* < 0, then we will randomly generate a point near the smallest one by Eq 11. Where, *x*_min_, *x*_*mid*_ and *x*_max_ are the resort points of *x*0, *x*1 and *x*2 according to their fitness separately. If a = 0, then the three points form a line. If a = 0 and b = 0, this means *y*0 = *y*1 = *y*2, then we will randomly select a position from the area, denoted as *x*3 (Eq 12). Where, $\frac{\left(x_{\max}+x_{\min}\right)}{2}$ is the search center, and because −0.5 < *rand* − 0.5 < 0.5, the random search radius is from 0 to half of the area. If a = 0 and b! = 0, then the solution will be chosen by Eq 11. When *I* = 0, which means *v*(*i*, *j*) = 0, then the procedure will be terminated.

$$\begin{array}{c} x_{3}=x_{\min}+rand*C_{3}*\left(x_{\min}-x_{mid}\right)+rand*C_{4}*\left(x_{\min}-x_{\max}\right) \end{array}$$ (11)

$$\begin{array}{c} x_{3}=\frac{\left(x_{\max}+x_{\min}\right)}{2}+\left(rand-0.5\right)\left(x_{\max}-x_{\min}\right) \end{array}$$ (12)

**Fig 2:**
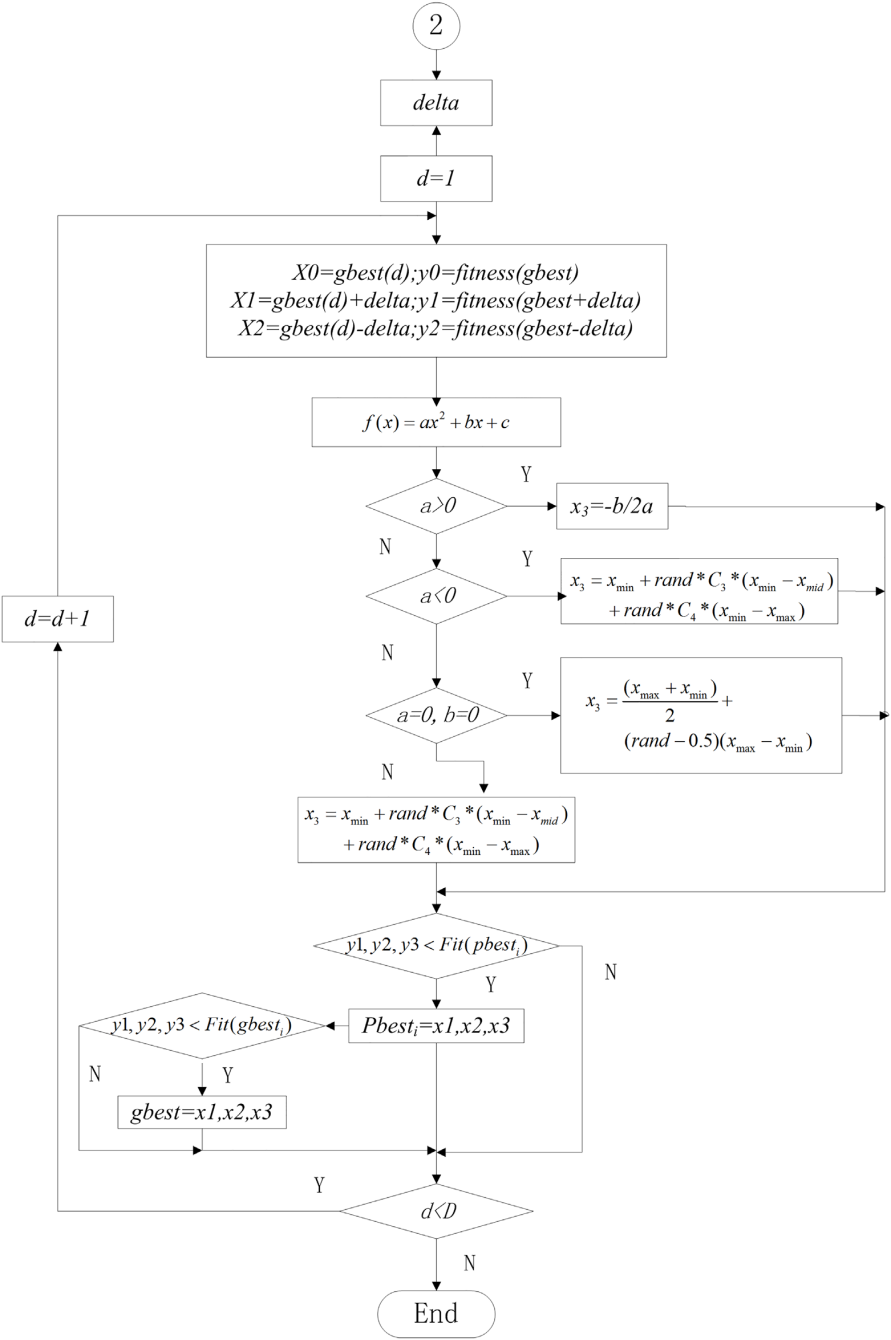
The flowchart of LSLI.

The flowchart of Lagrange interpolation is shown in Fig 2.

Comparing with other local search techniques, Lagrange interpolation has three characteristics. First, this method has a very fast convergence speed, especially for uni-modal functions. For example, for the Sphere function computation, whose global optima is [0, 0]^10^, supposing that the *gbest* is [1, 1]^10^, and the *delta* is 2. After one Lagrange interpolation computation, we obtain the next *gbest* is [0, 0]^10^. Second, for each dimension Lagrange interpolation, it will cost three additional FEs, and for D dimension problem, the additional FEs of the *gbest* will be 3*D. Third, the behaviour of Lagrange interpolation is only a local search, it will not broken the diversity of the whole particle swarm, hence it will remain the CLPSO’s search ability for multi-modal functions.

### 3.2 Lagrange interpolation learning (LIL)

In CLPSO, if the *i*^*th*^ particle’s *pbest* does not update for a certain number of times, then another two particles’ *pbest* will be chosen for comparison, and the better one will be the exemplar. However, if the exemplar is still worse than the *i*^*th*^ particle’s *pbest*, then this particle will remain stagnant until the next *flag*(*i*)>*m*, with a large probability. Hence, the best manner to improve the search efficiency is to set the *gbest* as the exemplar. Nevertheless, the so-called *gbest* in the computation is not the real *gbest* rather, it can be a best one of many local optima that the program runs until now. If we set *gbest* as the exemplar directly, as in the traditional PSO, then a premature convergence will occur.

**Fig 3:**
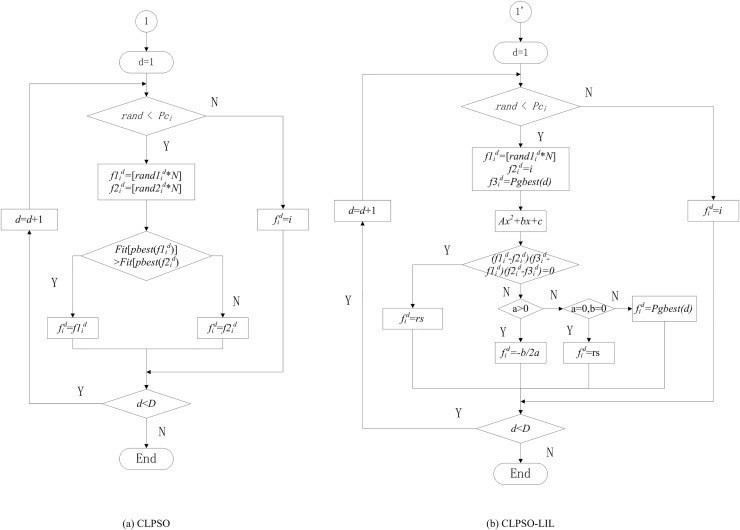
Selection of the exemplar dimensions for particle i. (a)CLPSO (b)CLPSO-LIL.

To avoid both the particle’s stagnant nature and premature convergence, we ensure that the exemplar’s information has two characteristics. First, the fitness of the exemplar is at least better than the *i*^*th*^ particle’s *pbest*. Second, the information from the exemplar has a diversity that cannot lead all the particles to prematurely fly into a same area. Hence, we decide to select three points to generate the information mentioned above. One point is the *i*^*th*^ particle’s *pbest* itself, another point is *gbest*, and the last point is the *rand*^*th*^ particle’s *pbest*, except for the *i*^*th*^ particle. In the *d*^*th*^ dimension space, the three points can generate a parabola by the Lagrange interpolation method, and the minimum point is desired. Fig 3 shows the difference between CLPSO and LIL.

This search behaviour has three strengths: First, the exemplar is the minimum from Lagrange interpolation with three points which are two *pbests* and one *gbest*, this process ensures the learning direction is always flying to a theoretical point which is better than *gbest*. Second, for most of the local search, additional time must be spent on computing the function evaluations (FEs) to obtain some key information, whereas LIL does not require any additional FEs. Third, after performing the LSLI mentioned in Section 3.1, we obtained a better *gbest*, then the LIL can share this information to other particles as soon as possible.

### 3.3 Parametric settings

To a convenient computation, we plan to run LSLI for N times, which is equal to the particle swarm size. However, To a fair comparison, noticing that there will be 3*D additional FEs for each LSLI, and we hope the total FEs of all compared algorithms are equal, thus we short the max iteration number (*max*_*gen*) to *max*_*gen* − 3 * *D*. The FEs cost in LSLI are 3*D*N, and FEs cost on other part are (*max*_*gen* − 3 * *D*)*N, plus them, we get the total FEs *max*_*gen* * *N*, which is the same with CLPSO.

When to run LSLI? we set a gap g, g = floor(maxDT/N), where, *maxDT* = *max*_*gen* − 3 * *D*. Hence, when the particle swarm updates for g times by Eqs 3 and 4, LSLI will run for one time.

In CLPSO, the learning probability pc(i) is set as Eq 5. In this research, we chose a linear probability form the Ref. [28] to increase the learning chance.

$$\begin{array}{c} pc(i)=0.05+0.45*i/N \end{array}$$ (13)

To compare with OLPSO [36], in this algorithm, we chose both c1 and c2 to be 2, w0 = 0.9 and w1 = 0.4. The setting of the bounds of the search space and the velocity of any particle affect the search procedure; hence we chose the same velocity boundary setting as that used in most of the algorithms.

$$V_{\max}=\alpha \left(x_{\max}-x_{\min}\right),\;\;\;\;V_{\min}=-V_{\max}$$

**Fig 4:**
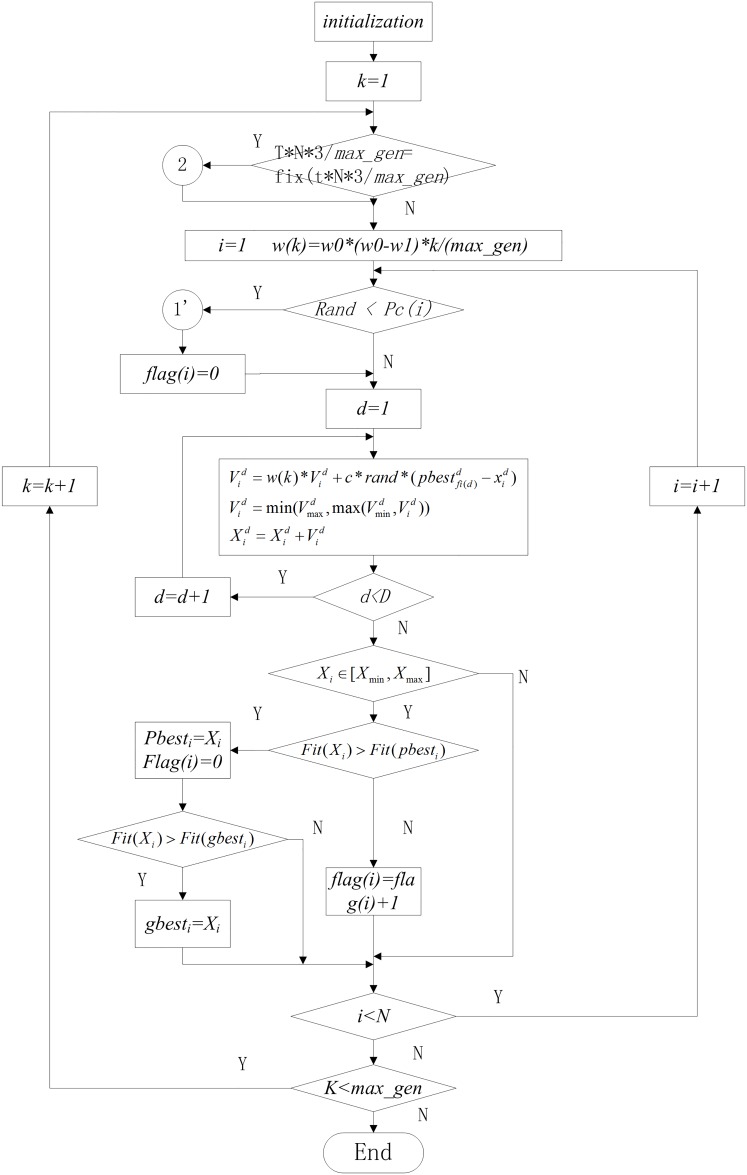
The flowchart of LILPSO.

Where, *α* = 0.2. The flow chart of LILPSO is shown in Fig 4.

## 4. Numerical experiments

Seventeen functions are collected from [17][19][37][38][39], as presented in Table 1, where, F1, F3, F4 and F5 are uni-modal functions; Rosenbrock (F2) is a multi-modal function that has a narrow valley and hard to achieve the global optimum; F5 is a noisy function who has a discrete problem; un-rotated multi-modal functions include F6 F11; F12 and F13 are rotated multi-modal functions; F14 F16 are shifted rotated multimodal functions. The orthogonal matrix M is generated according to [38].

**Table 1:** Details of benchmarks.

NO.	Func.name	Expression	Box constraint	optimum
F1	Sphere	$f\left(x\right)=\sum_{i=1}^{n}x_{i}^{2}$	[−100, 50]D	[0, 0]D
F2	Rosenbrock	$f\left(x\right)=\sum_{i=1}^{n-1}{100(x_{i+1}-x_{i}^{2})}^{2}+{\left(x_{i}-1\right)}^{2}$	[−30, 30]D	[0, 0]D
F3	Step	$f\left(x\right)=\sum_{i=1}^{n}{(x_{i}^{}+0.5)}^{2}$	[−100, 100]D	[0, 0]D
F4	Schwefel’s P2.22	$f\left(x\right)=\sum_{i=1}^{n}|x_{i}|+\prod_{i=1}^{n}|x_{i}|$	[−10, 10]D	[0, 0]D
F5	Noise Quadric	$f\left(x\right)=\sum_{i=1}^{n}ix_{i}^{4}+random\left(0,1\right)$	[−1.28, 1.28]D	[0, 0]D
F6	A generalized penalized	$\begin{array}{c} f\left(x\right)=\frac{\pi }{n}(10\sin^{2}\left(\pi y_{1}\right)\\ +\sum_{i=1}^{n}\left(y_{i}-1\right)\left(1+10\sin^{2}\left(\pi y_{i+1}\right)\right)+{\left(y_{n}-1\right)}^{2})\\ +\sum_{i=1}^{n}u(x_{i},10),\;\;\;\;\\ \;\;\;\;\;\;\;\;y_{i}=1+\left(x_{i}+1\right)/4,\;\;u\left(x_{i},a\right)\\ =\{\begin{array}{c} 100{\left(x_{i}-a\right)}^{4},\;\;if\;\;x_{i}>a\\ 0,\;\;if\;\;-a\leq x_{i}\leq a\\ 100{\left(-x_{i}-a\right)}^{4}\;\;if\;\;x_{i}<-a \end{array} \end{array}$	[−50, 50]D	[0, 0]D
F7	Another generalized penalized	$\begin{array}{c} f\left(x\right)=(\sin^{2}\left(3\pi x_{1}\right)+{\left(x_{n}-1\right)}^{2}(1+\sin^{2}\left(2\pi x_{n}\right)\\ \;\;\;\;\;\;\;+\sum_{i=1}^{n-1}{\left(x_{i}-1\right)}^{2}\left(1+\sin3\pi x_{i}\right))/10+\sum_{i=1}^{n}u(x_{i},5) \end{array}$	[−50, 50]D	[0, 0]D
F8	Ackley	$\begin{array}{c} f\left(x\right)=20+e-20*\exp\left(-0.2\sqrt{\frac{1}{n}\sum_{i=1}^{n}x_{i}^{2}}\right)\\ -\exp(\frac{1}{n}\sum_{i=1}^{n}\cos(2\pi x_{i})) \end{array}$	[−32, 32]D	[0, 0]D
F9	Rastrigin	$f\left(x\right)=\sum_{i=1}^{n}x_{i}^{2}-10\cos2\pi x_{i}+10$	[−5, 5]D	[0, 0]D
F10	Griewank	$f\left(x\right)=1+\frac{\sum_{i=1}^{n}{\left(x_{i}-100\right)}^{2}}{4000}-\prod_{i=1}^{n}\cos(\frac{x_{i}-100}{\sqrt{i}})$	[−600, 200]D	[0, 0]D
F11	Schwefel	$f\left(x\right)=418.9829-\sum_{i=1}^{n}x_{i}\sin\left(\sqrt{\left|x_{i}\right|}\right)$	[−500, 500]D	[420, 96]D
F12	Ackley-Rotated	*f*_12_(*y*) = *f*_8_(*y*), *y* = *Mx*	[−32, 32]D	[0, 0]D
F13	Rastrigin-Rotated	*f*_13_(*y*) = *f*_9_(*y*), *y* = *Mx*	[−5, 5]D	[0, 0]D
F14	Griewank-Rotated	*f*_14_(*y*) = *f*_10_(*y*), *y* = *Mx*	[−600, 200]D	[0, 0]D
F15	Ackley-Rotated-shifted	$f_{15}\left(y\right)=f_{8}\left(y\right)+f\_bias,\;\;y=M\left(\vec{x}-\vec{o}\right)$	[−32, 32]D	$\vec{o}$
F16	Rastrigin-Rotated-shifted	$f_{16}\left(y\right)=f_{8}\left(y\right)+f\_bias,\;\;y=M\left(\vec{x}-\vec{o}\right)$	[−5, 5]D	$\vec{o}$
F17	Griewank-Rotated-shifted	$f_{17}\left(y\right)=f_{10}\left(y\right)+f\_bias,\;\;y=M\left(\vec{x}-\vec{o}\right)$	[−600, 200]D	$\vec{o}$

Since some other algorithms, such as PSO-cf-local [36], UPSO [40], FIPS [41] and DMSPSO [42], are proven to be less superior to CLPSO in reference [19]; hence, in this research, we just need to compare LILCLPSO with CLPSO, ECLPSO, OLPSO and DNLPSO, whose iterative forms are presented in Table 2. The parametric setting of ECLPSO is the same as that of ECLPSO-4 in [22]. Because we do not know the exact orthogonal matrix for OLPSO, we introduce the result directly from the reference [19]. For DNLPSO, we don’t know the exact topology data, hence we introduce the result directly from the reference [26]. To proof the performance of LSLI and LIL, we test two algorithms, called LILPSO1 and LILPSO2, where, LILPSO1 just adapt LSLI technique, and LILPSO2 adapt both LSLI and LIL technique.

**Table 2:** Iterative forms of each algorithms.

Algorithm	Iterative forms
CLPSO [10]	*v*_*id*_(*t* + 1) = *ωv*_*id*_(*t*) + *c*_1_ *r*_1_(*p*_*id*_(*t*) − *x*_*id*_(*t*))
ECLPSO [33]	$v_{id}\left(t+1\right)=\omega v_{id}\left(t\right)+c_{1}r_{1}\left(p_{id}\left(t\right)+\eta \left(\frac{p_{d\_low}+p_{d\_up}}{2}-p_{id}\left(t\right)\right)-x_{id}\left(t\right)\right)$
OLPSO [38]	*v*_*id*_(*t* + 1) = *ωv*_*id*_(*t*) + *c*_1_ *r*_1_(*p*_*id*_(*t*) − *x*_*id*_(*t*))
DNLPSO [31]	*v*_*id*_(*t* + 1) = *ωv*_*id*_(*t*) + *c*_1_ *r*_1_(*p*_*id*_(*t*) − *x*_*id*_(*t*))+*c*_2_ *r*_2_(*p*_*gd*_(*t*) − *x*_*id*_(*t*))
LILPSO	*v*_*id*_(*t* + 1) = *ωv*_*id*_(*t*) + *c*_1_ *r*_1_(*p*_*id*_(*t*) − *x*_*id*_(*t*))

For 10 D problems, the following parameters are used: particle number = 50, iteration number = 2000, and FEs = 100,000; for 30 D problems, the following parameters are used: particle number = 40, iteration number = 5000, and FEs = 200,000; for 50 D problems, the following parameters are used: particle number = 100, iteration number = 5000, and FEs = 500,000. For each function, each algorithm runs for 25 times, and the solutions are analysed using the two-tailed t-test, with the confidence level of 0.05, ‘+’, ‘-’ and ‘=’ denote that LILPSO is better, worse and equal to other algorithms statistically, respectively.

**Fig 5:**
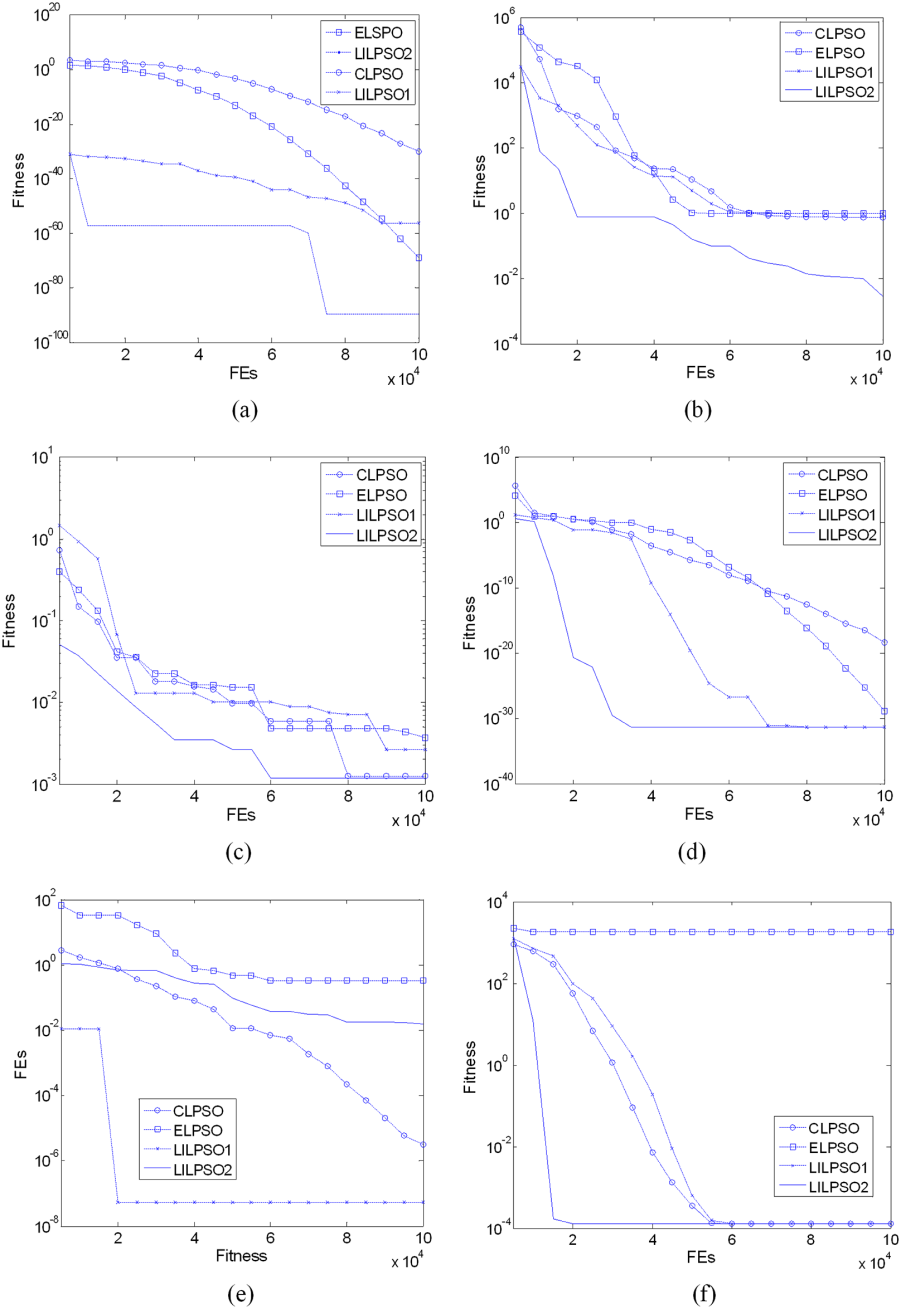
The comparison on convergence. (a) Sphere (b) Rosenbrock (c) Noise Quadric (d) Penalized (e) Griewank (f) Schwefel.

Tables 3–8 list the results tested in 10D, 30D and 50D, respectively, and Fig 5(a)-5(f) show these algorithms’ convergence curves for some different functions.

**Table 3:** Results for D = 10, N = 50, FEs = 100,000.

Function		Algorithm				
		CLPSO	ECLPSO	DNLPSO	LILPSO1	LILPSO2
F1	mean	4.94E−19	1.78E−30	**5.71E−177**	1.64E−58	1.11E−73
	sd	2.19E−37	2.34E−60	1.11E−123	7.11E−116	**6.27E−146**
	ttest	+	+	−	+	
F2	mean	1.26E+00	1	**1.86E−03**	1.00E+00	3.28E−01
	sd	1.79E+00	3.80E−09	2.64E−02	**6.32E−10**	1.71E−01
	ttest	+	+	−	+	
F3	mean	2.05E−18	1.03E−30	**0.00E+00**	**0.00E+00**	**0.00E+00**
	sd	2.27E−36	9.88E−61	**0.00E+00**	**0.00E+00**	**0.00E+00**
	ttest	+	+	=	=	
F4	mean	1.50E−11	6.22E+00	**1.96E−66**	2.29E−13	1.50E−16
	sd	3.73E−23	5.67E+01	**3.31E−54**	1.54E−27	1.61E−33
	ttest	+	+	−	+	
F5	mean	5.00E−03	2.30E−03	5.21E−01	4.50E−03	**1.40E−03**
	sd	3.34E−06	1.74E−06	1.89E−01	3.40E−06	**1.99E−07**
	ttest	+	+	+	+	
F6	mean	2.20E−18	2.85E−29	**4.71E−32**	**4.71E−32**	**4.71E−32**
	sd	4.46E−36	7.99E−57	**0.00E+00**	**0.00E+00**	**0.00E+00**
	ttest	+	+	=	=	
F7	mean	2.02E−16	1.41E−27	**1.35E−32**	2.45E−32	**1.35E−32**
	sd	3.24E−32	5.91E−54	**0.00E+00**	1.44E−65	**0.00E+00**
	ttest	+	+	=	=	
F8	mean	1.94E−08	3.55E−15	**3.26E−15**	2.58E−11	1.84E−14
	sd	1.49E−16	4.21E−30	1.83E−15	3.02E−22	**6.84E−28**
	ttest	+	+	=	+	
F15	mean	3.76E−02	2.31E+00	2.02E+01	6.27E−05	**2.01E−08**
	sd	4.01E−04	2.83E+01	4.04E−01	4.38E−09	**2.26E−16**
	ttest	+	+	+	+	
F16	mean	6.11E+00	7.09E+01	7.57E+00	2.71E+00	**2.67E+00**
	sd	7.02E+00	1.03E+02	**4.70E+00**	8.83E+00	6.65E+00
	ttest	+	+	+	+	
F17	mean	1.40E−01	1.84E+01	**8.07E−03**	1.52E−04	9.29E−02
	sd	3.10E−03	2.53E+02	1.14E−01	**2.01E−08**	1.09E−02
	ttest	+	+	=	−	

**Table 4:** Results for D = 10, N = 50, FEs = 100,000.

Function		Algorithm			
		CLPSO	ECLPSO	LILPSO1	LILPSO2
F9	mean	**0.00E+00**	**0.00E+00**	**0.00E+00**	**0.00E+00**
	sd	**0.00E+00**	**0.00E+00**	**0.00E+00**	**0.00E+00**
	ttest	=	=	=	
F10	mean	**7.92E−05**	3.16E−01	1.26E−04	5.00E−03
	sd	**4.09E−08**	1.33E−02	6.90E−08	5.15E−05
	ttest	−	+	−	
F11	mean	**1.27E−04**	1.78E+03	**1.27E−04**	**1.27E−04**
	sd	6.34E−26	3.26E+04	**0.00E+00**	**0.00E+00**
	ttest	=	+	=	
F12	mean	3.08E−02	3.66E+00	2.46E−06	**2.87E−08**
	sd	1.38E−04	4.42E+01	2.33E−12	**6.09E−16**
	ttest	+	+	+	
F13	mean	6.41E+00	6.15E+01	**1.35E+00**	6.95E+00
	sd	4.49E+00	8.91E+01	**2.16E+00**	5.45E+01
	ttest	−	+	−	
F14	mean	1.64E−01	1.06E+01	**6.27E−05**	5.34E−02
	sd	6.20E−03	1.08E+02	**4.38E−09**	5.80E−03
	ttest	+	+	+	

**Table 5:** Results for D = 30, N = 40, FEs = 200,000.

Function		Algorithm					
		CLPSO	ECLPSO	OLPSO-G	OLPSO-L	LILPSO1	LILPSO2
F1	mean	5.66E−15	1.32E−26	4.12E−54	1.11E−38	3.00E−66	**4.19E−87**
	sd	1.33E−29	1.63E−51	6.34E−54	1.28E−38	4.31E−131	**8.79E−173**
	ttest	+	+	+	+	+	
F2	mean	8.28E+00	**1.00E+00**	2.15E+01	1.26E+00	**1.00E+00**	**1.00E+00**
	sd	3.57E+01	**0.00E+00**	2.99E+01	1.40E+00	2.39E−13	1.90E−05
	ttest	+	=	+	+	=	
F4	mean	3.72E−09	7.41E+01	**9.85E−30**	7.67E−22	4.81E−13	4.86E−17
	sd	2.12E−18	1.33E+02	1.01E−29	5.63E−22	8.61E−27	**1.83E−33**
	ttest	+	+	−	−	+	
F5	mean	1.57E−02	**6.40E−03**	1.16E−02	1.64E−02	1.05E−02	8.10E−03
	sd	1.31E−05	5.48E−06	4.10E−03	3.25E−03	1.65E−05	**4.53E−06**
	ttest	+	=	+	+	+	
F6	mean	1.63E−15	6.81E−22	1.59E−32	**1.57E−32**	**1.57E−32**	**1.57E−32**
	sd	1.05E−30	6.94E−42	1.03E−33	2.79E−48	**0.00E+00**	**0.00E+00**
	ttest	+	+	=	=	=	
F7	mean	2.05E−12	5.83E−20	4.39E−04	1.57E−32	1.33E−31	**1.35E−32**
	sd	3.59E−24	5.47E−38	2.20E−03	2.79E−48	1.90E−62	**0.00E+00**
	ttest	+	+	+	=	+	
F8	mean	6.92E−07	2.20E−05	7.98E−15	4.14E−15	2.25E−11	**2.84E−15**
	sd	1.21E−13	9.75E−09	2.03E−15	**0.00E+00**	5.35E−22	2.52E−30
	ttest	+	+	+	+	+	
F9	mean	1.77E−15	1.91E+00	2.17E+02	**0.00E+00**	**0.00E+00**	**0.00E+00**
	sd	0.00E+00	6.95E+01	1.07E+00	**0.00E+00**	**0.00E+00**	**0.00E+00**
	ttest	+	+	+	=	=	
F10	mean	3.35E−10	1.88E+02	4.83E−03	**0.00E+00**	**0.00E+00**	**0.00E+00**
	sd	4.02E−19	1.15E+03	8.63E−03	**0.00E+00**	**0.00E+00**	**0.00E+00**
	ttest	+	+	+	=	=	
F11	mean	3.81E−04	6.58E+03	3.84E+02	**3.81E−04**	2.36E+01	**3.81E−04**
	sd	3.41E−23	2.53E+05	2.17E+02	**0.00E+00**	2.80E+03	**0.00E+00**
	ttest	=	+	+	=	+	
F12	mean	3.36E+00	1.83E+01	7.69E−15	**4.28E−15**	6.40E−03	2.60E−03
	sd	5.73E−01	1.59E−01	1.78E−15	**7.11E−16**	6.49E−15	9.19E−06
	ttest	+	+	−	−	+	
F13	mean	3.92E+01	3.15E+02	**4.60E+00**	5.34E+01	2.92E+01	3.56E+01
	sd	5.80E+01	4.78E+02	**1.28E+01**	1.33E+01	9.63E+01	6.96E+02
	ttest	+	+	−	+	−	
F14	mean	1.06E+00	2.09E+02	1.68E−03	**4.19E−08**	7.69E−04	7.06E−02
	sd	2.10E−03	8.32E+01	4.13E−03	**2.06E−07**	8.66E−07	7.70E−03
	ttest	+	+	−	−	−	

**Table 6:** Results for D = 30, N = 40, FEs = 200,000.

Function		Algorithm			
		CLPSO	ECLPSO	LILPSO1	LILPSO2
F3	mean	1.41E−14	1.13E−19	**0.00E+00**	**0.00E+00**
	sd	4.76E−29	2.53E−37	**0.00E+00**	**0.00E+00**
	ttest	+	+	=	
F15	mean	3.24E+00	1.87E+01	3.60E−03	**1.10E−03**
	sd	3.78E−01	2.50E−01	2.81E−05	**2.60E−06**
	ttest	+	+	+	
F16	mean	3.54E+01	3.53E+02	**2.36E+01**	4.66E+01
	sd	2.25E+01	8.27E+02	**2.19E+01**	7.91E+01
	ttest	−	+	=	
F17	mean	1.06E+00	2.15E+02	**1.40E−02**	3.46E−02
	sd	4.70E−03	8.67E+01	**7.11E−04**	4.20E−03
	ttest	+	+	−	

**Table 7:** results for D = 50, N = 100, FEs = 500,000.

Function		Algorithm				
		CLPSO	ECLPSO	DNLPSO	LILPSO1	LILPSO2
F1	mean	1.07E−08	8.06E+03	9.44E−74	2.60E−57	**4.77E−74**
	sd	5.82E−18	1.26E+07	9.13E−32	1.22E−113	**5.61E−147**
	ttest	+	+	+	+	
F2	mean	3.16E+01	1.08E+01	**1.86E−03**	1.00E+00	2.28E−01
	sd	2.04E+02	8.00E+02	2.64E−02	**6.23E−14**	1.89E−01
	ttest	+	+	−	+	
F3	mean	3.19E−08	2.03E+04	**0.00E+00**	**0.00E+00**	**0.00E+00**
	sd	4.15E−17	1.14E+08	**0.00E+00**	**0.00E+00**	**0.00E+00**
	ttest	+	+	=	=	
F4	mean	2.59E−05	1.27E+02	7.02E−09	8.85E−09	**3.84E−13**
	sd	2.80E−11	2.38E+02	2.29E−06	1.99E−18	**5.09E−25**
	ttest	+	+	+	+	
F5	mean	2.69E−02	1.97E−02	6.11E−01	1.32E−02	**9.30E−03**
	sd	1.64E−05	2.05E−05	2.20E−01	**1.92E−06**	8.08E−06
	ttest	+	+	+	+	
F6	mean	1.73E−09	1.35E+02	1.50E−32	1.28E−32	**9.42E−33**
	sd	3.25E−19	3.25E+05	0.00E+00	2.57E−66	**0.00E+00**
	ttest	+	+	+	+	
F7	mean	1.69E−06	1.92E+02	1.34E−32	8.75E−31	**9.42E−33**
	sd	2.23E−13	7.26E+05	0.00E+00	1.77E−60	**0.00E+00**
	ttest	+	+	+	+	
F8	mean	3.95E−04	1.58E+01	1.90E+01	8.13E−08	**3.05E−14**
	sd	1.20E−08	1.43E+01	0.00E+00	1.87E−15	**1.55E−27**
	ttest	+	+	+	+	
F15	mean	6.80E+00	1.81E+01	2.07E+01	**1.86E−01**	3.07E−01
	sd	2.47E−01	9.77E−02	1.13E−02	**2.86E−02**	3.61E−01
	ttest	+	+	+	−	
F16	mean	1.07E+02	1.65E+02	**3.40E+01**	8.83E+01	8.13E+01
	sd	5.02E+01	2.03E+02	**8.03E+00**	2.27E+02	1.22E+04
	ttest	+	+	−	=	
F17	mean	2.63E+00	4.40E+02	**1.11E−02**	2.75E−01	2.54E−01
	sd	1.61E−01	4.54E+01	**1.12E−02**	2.46E−02	4.04E−02
	ttest	+	+	−	=	

**Table 8:** results for D = 50, N = 100, FEs = 500,000.

Function		Algorithm			
		CLPSO	ECLPSO	LILPSO1	LILPSO2
F9	mean	1.22E−10	6.80E+01	**0.00E+00**	**0.00E+00**
	sd	7.80E−22	4.54E+02	**0.00E+00**	**0.00E+00**
	ttest	+	+	=	
F10	mean	2.56E−07	5.71E+02	**0.00E+00**	**0.00E+00**
	sd	1.34E−14	3.73E+04	**0.00E+00**	**0.00E+00**
	ttest	+	+	=	
F11	mean	6.59E−04	1.44E+04	**6.36E−04**	**6.36E−04**
	sd	1.11E−10	2.77E+06	2.53E−17	**0.00E+00**
	ttest	+	+	=	
F12	mean	8.12E+00	1.74E+01	**7.88E−02**	4.22E+00
	sd	9.58E−01	4.08E−01	**1.50E−03**	7.79E+01
	ttest	+	+	−	
F13	mean	1.09E+02	4.89E+02	**9.51E+01**	1.12E+02
	sd	1.07E+02	9.71E+02	8.31E+01	**2.64E+03**
	ttest	−	=	−	
F14	mean	2.54E+00	4.33E+02	**2.28E−01**	2.88E−01
	sd	9.47E−02	8.61E+01	**3.30E−03**	4.00E−02
	ttest	+	+	−	

For the uni-modal and low dimension problems, DNLPSO has the better performance relatively. For the noisy function F5, LILPSO has the better performance. For uni-modal function F3, DNLPSO and LILPSO have the equal solutions. Because DNLPSO’s iterative form contains the gbest part, it will have a fast convergence for uni-modal functions without a doubt. Besides, the neighbourhoods topology behaviour plays a role of decreasing the search space actually. However, for the high dimension problems, i.e. 50D, LILPSO has all the best solutions, which illustrates the Lagrange interpolation technique has a fast convergence performance for complex high dimension problems.

For the multi-modal problems, DNLPSO is less superior to LILPSO for almost all the functions. Comparing OLPSO with LILPSO in 30D problems, for F2 and F8, LILPSO has the better solutions; for F6, F7, F9, F10 and F11, LILPSO has the equal solutions with OLPSO statistically, which illustrate that the Lagrange interpolation technique can help accelerating the pbest’s convergence when performing a local search. For F12, F13 and F14, OLPSO is superior to LILPSO, which illustrates that LSLI is restricted to rotated problems, although it is still better than CLPSO.

Comparing LILPSO1 with LILPSO2, for the most problems, LILPSO2 is superior to LILPSO1, which illustrates that LIL can help sharing the LSLI’s information to accelerate the convergence. Meanwhile, unlike the gbest’s part of DNLPSO, LIL neither break the diversity of the particle, nor lead the particle to premature. However, for the rotated functions such as F13, F14, F16 and F17, the solutions of LILPSO1 are better than LILPSO2, which illustrates that LIL is not suitable for solving the rotated problems either.

Comparing CLPSO with LILPSO, LILPSO has all the better solutions than CLPSO, which illustrates that the Lagrange interpolation is a stable and efficient local search technique.

Comparing OLPSO with LILPSO, they both inherit the advantage of CLPSO in solving the multi-modal problems, nevertheless, LILPSO is obviously superior to OLPSO in solving the uni-modal problems. Moreover, OLPSO-L needs O(2^log_2_ (*D* + 1)^ *D* + *ND*) memory space to store its algorithm related data structures, which means longer cost time for complex real world problems. In contrast, LILPSO just needs a small number of memory space to store some related variables, i.e. x0, y0, x1, y1, x2, y2, a, b and c.

## 5. Application for PID control

The fan speed system controlled by oil in air turbofan launch is taken for example. The transfer function model of the system is:

$$\frac{1.192s+6.273}{s^{2}+7.167s+12.84}$$

PID discretion control equation is

$$Kp*e\left(k\right)+Ki*\sum_{i=0}^{k}e(i)+Kd\left[e\left(k\right)-e\left(k-1\right)\right]$$

The objective function is:

$$T=\int_{0}^{\infty }(\omega 1\cdot \left|e(t)\right|+\omega 2\cdot u^{2}\left(t\right))dt+\omega 3\cdot t_{u}$$

Where, |*e*(*t*)| is the error, *t*_*u*_ is the rise time, *u*(*t*) is the output of the controller, *ω*1, *ω*2, *ω*3 are the weight. To solve the problem of system overshoot, use the punish function, once the system overshoot happens, the objective function will be:

$$T=\int_{0}^{\infty }(\omega 1\left|e(t)\right|+\omega 2u^{2}\left(t\right)+\omega 4\left|ey(t)\right|)dt+\omega 3\cdot t_{u}$$

Where, *ω*4 > >*ω*1, *ey*(*t*) = *y*(*t*) − *y*(*t* − 1), *y*(*t*) is the output of the object controlled. In this example, *ω*1 = 0.5, *ω*2 = 1, *ω*3 = 1, *ω*4 = 200, the range of Kp, Ki, Kd is respectively [0.2, 10], [1, 50], [1e-7, 1e-1], and the overshoot should be smaller than 20%. Hence, the problem is:

$$\begin{array}{c} \begin{array}{c} \min\;\;\;\;T\\ s.t.\;\;\;\;\;\;0.2\leq Kp\leq 10\\ \;\;\;\;\;\;\;\;\;1\leq Ki\leq 50\\ \;\;\;\;\;\;\;\;\;1e-7\leq Kd\geq 1e-1\\ \;\;\;\;\;\;\;\;\;overshoot\leq 0.2 \end{array} \end{array}$$ (14)

The parameters are set as: swarm size N = 30, function evaluations FEs = 3000. The results are shown in Table 9. It illustrates that the results optimized by LILPSO2 have the smallest goal function value, and the mean error. Hence, LILPSO2 algorithm is more efficient.

**Table 9:** PID Results optimized by some algorithms.

Variables	CLPSO [10]	ECLPSO [33]	LILPSO2
*Kp*	3.0934	2.719	4.402
*Ki*	17.2368	16.817	24.58
*Kd*	0.042	6.13E−05	0.0964
*overshoot*	10.79%	11.75%	9.90%
*Mean error*	0.0099	0.0101	0.0079
*T*	2261	2267	2218

## 6. Conclusions

In this study, we proposed a novel method known as LILPSO to improve upon the state-of-the-art CLPSO method. First, the Lagrange interpolation approach is introduced to perform a local search near the gbest, and help accelerating convergence. Second, this technique is introduced to replace the simple comparison used in CLPSO, to achieve a better exemplar. After performing numerical experiments, LILPSO was proven to be superior to CLPSO, ECLPSO, DNLPSO, OLPSO for most of the test functions considered. The Lagrange interpolation was proven to be an efficient local search approach except for rotated problems. The future work is to use this method into other fields.

## Supporting Information

S1 Table The comparison results for CLPSO, ECLPSO, DNLPSO, LILPSO1 and LILPSO2. (XLS)
